# Sphingolipids associated research in Alzheimer’s disease: an explored trends analysis

**DOI:** 10.3389/fnagi.2026.1723883

**Published:** 2026-06-09

**Authors:** Ming-Rong Xie, Yan-Jun Chen, Hui Yuan

**Affiliations:** 1 The First Clinical Medical College, Nanjing University of Chinese Medicine, Nanjing, China; 2 Hunan University of Chinese Medicine, Changsha, China; 3 Department of Chinese Medicine, Hunan Aerospace Hospital, The Affiliated Aerospace Hospital of Hunan Normal University, Changsha, China

**Keywords:** Alzheimer’s disease, lipidomics, metabolomics, microglia, sphingolipids

## Abstract

**Background:**

Alzheimer’s disease (AD) is a progressive neurodegenerative disorder characterized by cognitive impairment. Numerous studies have indicated that dysregulation of sphingolipid metabolism is closely associated with the core pathology of AD and acts as a crucial driving factor. This study aims to employ bibliometric methods to comprehensively summarize the relevant research on sphingolipids in AD, identify research hotspots and emerging trends, and thereby provide objective data to guide future research directions.

**Methods:**

Publications were retrieved from the Web of Science and Scopus databases. Visual analyses were performed using CiteSpace, VOSviewer, and Bibliometrix.

**Results:**

A total of 623 publications related to sphingolipids and AD were included. The United States and China were the leading contributors in this field. Johns Hopkins University was the most prolific institution. *Journal of Alzheimer’s Disease* was the most frequently published journal. Dr. Erhard Bieberich was the author with the highest number of publications. High-frequency keywords included AD, sphingolipids, sphingolipid metabolism, ceramide, sphingomyelin, and cholesterol. Keywords with the strongest bursts in recent years included lipidomics, metabolomics, microglia, and cognitive impairment.

**Conclusion:**

Research on sphingolipids in AD exhibits an overall fluctuating upward trend. Extensive collaboration among researchers from various institutions has facilitated advancements in this field. Sphingolipid metabolism represents a key focus in AD research, with ceramides and sphingomyelins identified as critical molecules. Lipidomics, metabolomics, and microglia are likely to represent future research frontiers.

## 1 Introduction

Alzheimer’s disease (AD) is a progressive neurodegenerative disorder characterized by cognitive impairment and behavioral changes, which imposes a substantial economic and care burden on society. It’s typical neuropathological features include extracellular senile plaques formed by amyloid β (Aβ) deposition and intracellular neurofibrillary tangles composed of hyperphosphorylated Tau protein (Querfurth and LaFerla, 2010). The mainstream amyloid cascade hypothesis posits that an imbalance between the abnormal production and clearance of Aβ is the initiating event in AD pathogenesis, while Tau pathology, neuroinflammation, and synaptic damage represent key downstream events (Hardy and Higgins, 1992; Long and Holtzman, 2019). However, the pathogenesis of AD is highly complex, with many regulatory pathways yet to be fully elucidated. Therefore, moving beyond conventional research on Aβ and Tau to explore novel molecular mechanisms is crucial for revealing the complete pathogenic picture of AD, developing early diagnostic biomarkers, and formulating effective treatment strategies.

Sphingolipids are a class of complex lipids with sphingosine as the backbone, serving as key structural components of the myelin sheaths in the central nervous system and neuronal membranes. Ceramide, the core structure of sphingolipids, acts as a common precursor and central node in the biosynthesis of various sphingolipid species (Futerman and Hannun, 2004). Sphingolipids are primarily classified into sphingomyelin and glycosphingolipids (Merrill, 2011). Under physiological conditions, sphingolipids, particularly sphingomyelin and cholesterol, form dynamic microdomains on the cell membrane known as lipid rafts. These lipid rafts serve as platforms that organize signal transduction complexes, ensuring the specificity and efficiency of signal transmission (Simons and Ikonen, 1997). Moreover, sphingolipid metabolites themselves function as important signaling molecules. Ceramide is involved in the regulation of cellular stress, apoptosis, and inflammation (Maceyka and Spiegel, 2014), whereas its derivative, sphingosine-1-phosphate, promotes cell survival, proliferation, and migration. These metabolites form a sphingolipid rheostat that dynamically balances cell fate (Hannun and Obeid, 2008). Under pathological conditions, aberrant sphingolipid metabolism is associated with various disorders, including sphingolipid storage diseases caused by enzymatic deficiencies, insulin resistance, and neurodegenerative diseases (Park et al., 2016; Futerman and van Meer, 2004; Hannun and Obeid, 2018).

Numerous studies have demonstrated that disruptions in sphingolipid metabolism are closely intertwined with the core pathology of AD and play a critical driving role. Both β-secretase (BACE1) and γ-secretase complexes are highly enriched in sphingolipid-rich lipid rafts (He et al., 2010). Elevated levels of sphingolipids/ceramides in the cell membrane have been shown to enhance BACE1 activity and promote its co-localization with amyloid precursor protein (APP), thereby driving Aβ production (Cordy et al., 2003). Sphingolipids also directly facilitate Aβ aggregation and toxicity. Specific glycosphingolipids (such as GM1) can interact with Aβ to form GAβ complexes, accelerating Aβ fibrillization and senile plaque formation (Yanagisawa, 2007). Thus, sphingolipid metabolism constitutes an indispensable aspect of AD research, and a detailed understanding of its mechanisms will provide crucial new insights for overcoming this complex disease.

Bibliometrics is a discipline that employs mathematical and statistical methods to quantitatively analyze academic literature and its related characteristics—such as publication volume, authors, institutions, keywords, and citation networks. Applying bibliometric analysis to the study of sphingolipids in AD transcends the subjective limitations of traditional reviews. Through the use of visual mapping and data modeling, it objectively and macroscopically reveals the evolution of the field, core research themes, key scholars, collaborative networks, and emerging trends. This approach provides robust, data-driven support, enabling researchers to quickly grasp the overall landscape, identify foundational knowledge, and optimize decisions regarding scientific collaboration.

## 2 Materials and methods

### 2.1 Data search

**FIGURE 1:**
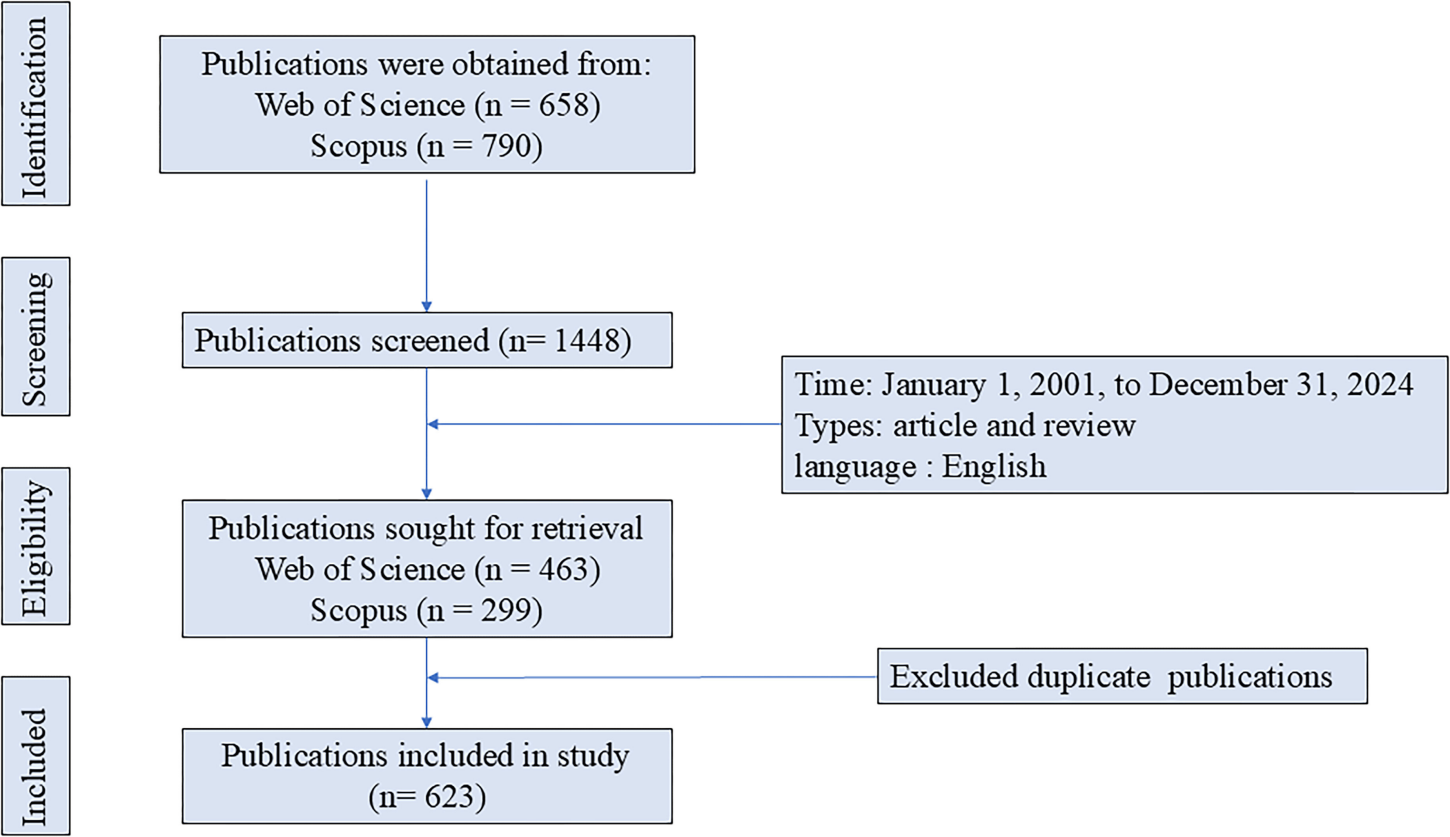
Flowchart of publication search process.

Relevant publications on sphingolipids and AD were retrieved from the Web of Science (WoS) and Scopus databases. The WoS search formula was: ((TS = (Sphingolipid)) OR TS = (Sphingolipids)) AND ((((TS = (Alzheimer’s disease) OR TS = (Alzheimer disease)) OR TS = (alzheimers-disease)) OR TS = (Alzheimer Diseases)) OR TS = (Alzheimers Diseases)). The Scopus search formula was: (TITLE-ABS-KEY (Sphingolipid OR Sphingolipids) AND TITLE-ABS-KEY (Alzheimer’s disease OR Alzheimer disease OR alzheimers-disease OR Alzheimer Diseases OR Alzheimers Diseases)). The search period was set up to December 31, 2024, with language restricted to English and publication types limited to reviews and articles. Two researchers independently screened the included publications and excluded those not relevant to the research topic, as well as duplicate records. Ultimately, a total of 623 publications related to sphingolipids and AD were identified (Figure 1).

### 2.2 Data analysis

The selected publications were visually analyzed using CiteSpace, VOSviewer, and Bibliometrix software, following methodologies consistent with previous studies (Chen et al., 2025a; Chen et al., 2025b). CiteSpace enables the visualization of scientific knowledge maps over time, focusing on the evolution of research fronts, detection of emerging topics, and identification of transitions between research paradigms, thereby offering historical and predictive insights (Chen, 2004). VOSviewer features powerful graph layout and clustering algorithms, allowing clear visualization of co-occurrence or collaboration networks among authors, institutions, keywords, or journals (van Eck and Waltman, 2010). Bibliometrix is capable of analyzing research outcomes, citation patterns, and scientific networks. It achieves in-depth data mining through integrated statistical models and data processing (Aria and Cuccurullo, 2017).

### 3 Results

### 3.1 Publication trends

**FIGURE 2:**
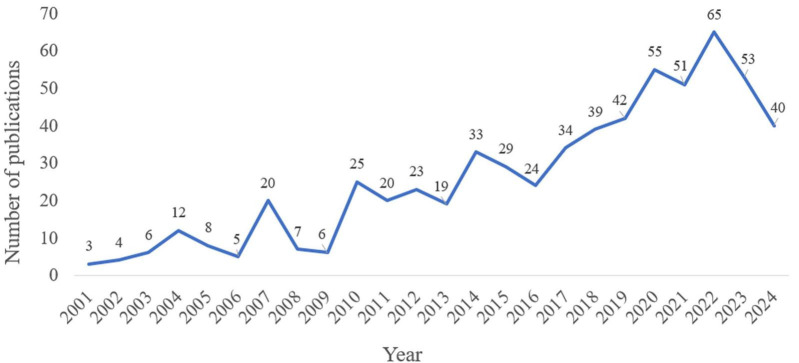
Annual number of publications on Sphingolipids and AD.

The development trend of sphingolipids in the field of AD research displayed a distinct three-stage pattern (Figure 2). Initial exploration stage (2001–2009): Before 2010, the number of publications was relatively small, with slow growth. Research on the association between sphingolipids and AD was still a niche area and had not yet garnered broad academic interest. This period likely represented a formative phase of theoretical proposal and preliminary validation. Fluctuating growth period (2010–2016): Beginning in 2010, the annual publication count entered a phase of relatively stable growth. This turning point reflected the scientific community’s growing recognition of the significant role sphingolipids play in AD pathology. Growth phase (2017–2024): After 2017, the number of publications exhibited an exponential increase. The growth rate accelerated notably after 2020, peaking at approximately 65 publications in 2022. This surge may be attributed to advances in lipidomics technologies and increasing academic focus on the roles of neuroinflammation and metabolic factors in disease.

### 3.2 Country

**FIGURE 3:**
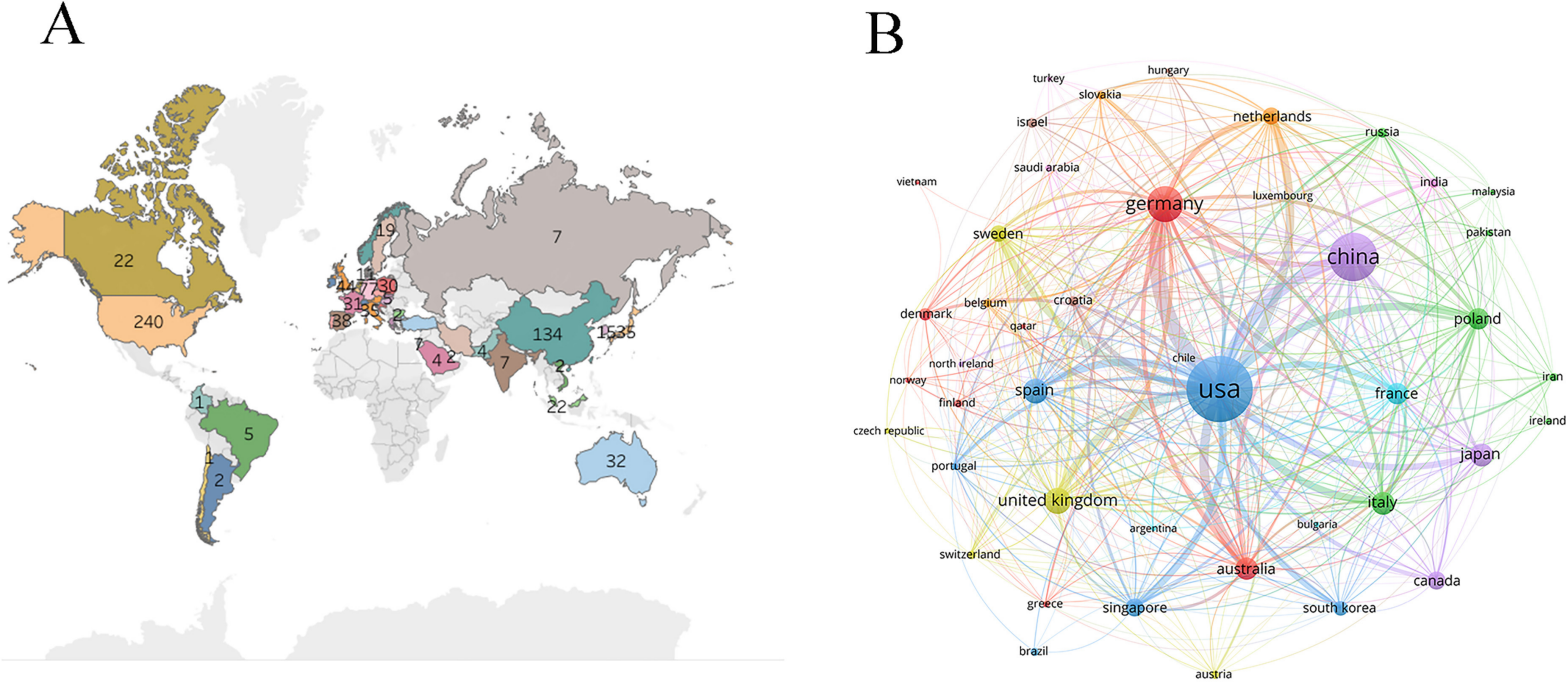
Country analysis. **(A)** Geographic distribution of publications. **(B)** International collaborative network.

Analysis of global research contributions revealed a high degree of concentration, with the United States (240 publications) and China (134 publications) leading the field (Figure 3A). The output from these two nations substantially surpasses that of all other countries, jointly establishing the global first echelon and core research influence in this area (Table 1). Both the United States and China also occupied central positions within the collaborative network and maintain numerous international partnerships (Figure 3B). The structure of international collaboration was not uniformly distributed but exhibited clear clustering. The United States maintained strong collaborative ties with Germany, China, Spain, Canada, Singapore, Australia, and others, forming an extensive international collaboration network. This underscored the United States not only as a high-output contributor but also as a global hub for collaborative research. European nations, including the United Kingdom, Germany, France, Italy, Spain, and the Netherlands, exhibited dense interconnections, forming another robust collaborative cluster. This reflected a tradition of close scientific collaboration within the European Union framework.

**TABLE 1:** The top 10 countries.

Rank	Country	Number of publications	Citations	Total link strength
1	USA	240	1,6252	2,681
2	China	134	3,865	747
3	Germany	77	4,951	1,151
4	United Kingdom	44	2,211	522
5	Spain	38	1,200	361
6	Italy	35	1,664	395
7	Japan	35	1,664	275
8	Australia	32	1,567	527
9	France	31	1,611	345
10	Poland	30	958	405

### 3.3 Institutions

**FIGURE 4:**
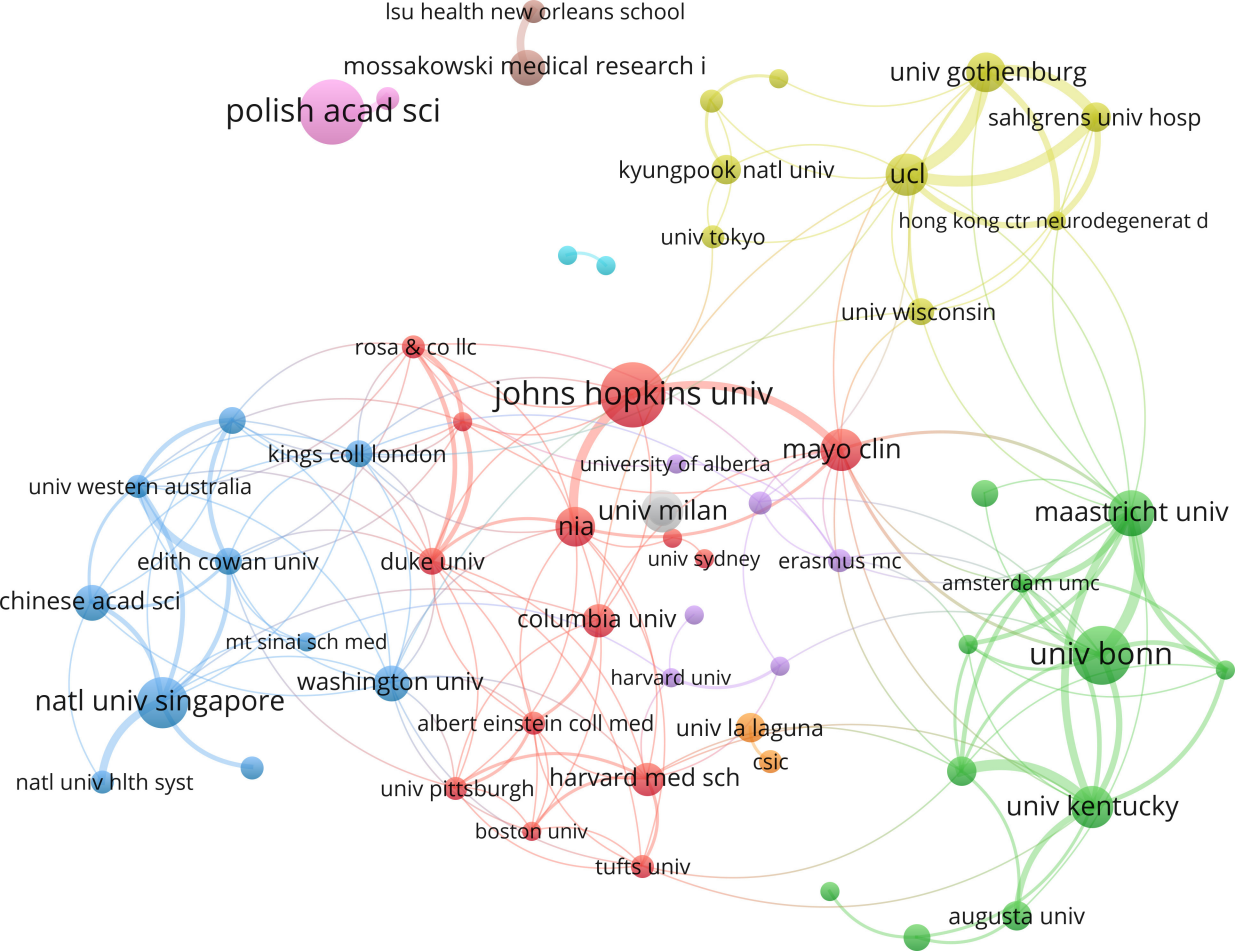
Institutional collaborative network.

Johns Hopkins University (19 publications, 2,991 citations) was undoubtedly a leader, ranking first in both output and overall influence. Although the National Institute on Aging (NIA) has published 10 articles, its total citations reach 1,813, with an average of 181.3 citations per article, far exceeding those of other institutions (Table 2). This clearly indicated that the NIA has published several landmark papers in this field, demonstrating its absolute authority and influence. Research institutions worldwide have formed a multicenter collaborative network, with institutions in the United States and Europe occupying central positions (Figure 4). Leading institutions included top comprehensive universities (such as Johns Hopkins University and the University of Bonn), renowned medical centers/hospitals (such as Mayo Clinic and Massachusetts General Hospital), and national research institutions (such as NIA and the Polish Academy of Sciences). This reflected that research on sphingolipids in AD required deep integration of multiple disciplines, including basic neuroscience, clinical medicine, and public health.

**TABLE 2:** The top 10 institutions.

Rank	Institution	Documents	Citations	Average number of citations
1	Johns Hopkins University	19	2,991	157.42
1	Polish Academy of Sciences	19	910	47.89
3	University of Bonn	17	966	56.82
4	National University of Singapore	14	869	62.07
5	Maastricht University	12	364	30.33
6	Mayo clinic	11	988	89.82
6	University College London	11	415	37.73
6	University of Kentucky	11	202	18.36
6	University of Milan	11	600	54.55
10	NIA	10	1813	181.30
10	University of Gothenburg	10	386	38.60

### 3.4 Journals

**FIGURE 5:**
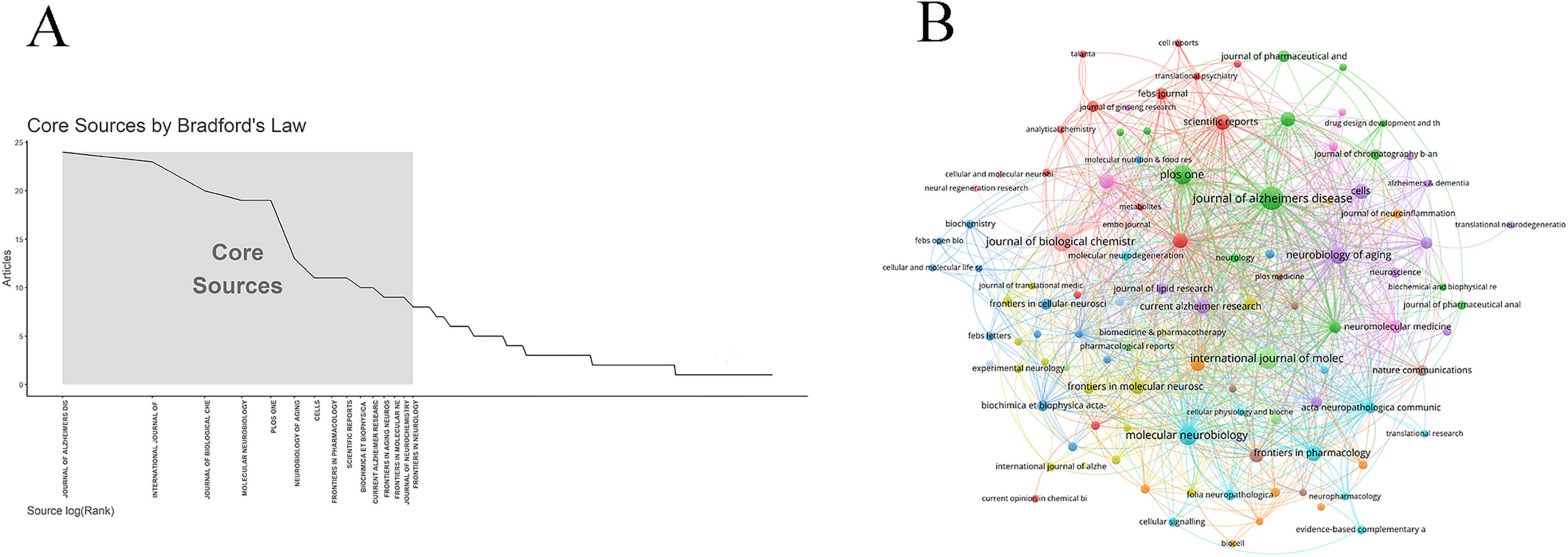
Journal analysis. **(A)** Core journal. **(B)** Journal network.

Based on Bradford’s law, 15 core journals closely related to the research topic were identified (Figure 5A). Research findings in this field were mainly published in three major types of journals, forming a stable dissemination system (Figure 5B). Neuroscience/AD journals: such as the *Journal of Alzheimer’s Disease* (31 publications), *Neurobiology of Aging*, and *Current Alzheimer’s Research*. These journals served as the primary outlets for research results, directly targeting the intended audience. Comprehensive/multidisciplinary science journals: such as the *International Journal of Molecular Sciences* (23 publications), *PLOS ONE*, and *Scientific Reports*. These journals published a large volume of papers and covered a broad scope, reflecting the wide attention and interdisciplinary nature of this research field. Core biochemistry/molecular biology journals: such as the *Journal of Biological Chemistry* (20 publications) and *Biochimica et Biophysica Acta - Molecular and Cell Biology of Lipids* (Table 3). These journals often emphasized fundamental mechanisms and biochemical depth.

**TABLE 3:** The top 10 journals.

Rank	Source	Documents	Citations	Average number of citations	IF	JCR
1	Journal of Alzheimer’s Disease	31	1,185	38.23	3.1	Q2
2	International Journal of Molecular Sciences	23	1,263	54.91	4.9	Q1
3	Journal of Biological Chemistry	20	2,698	134.90	3.9	Q2
4	Molecular Neurobiology	19	878	46.21	4.3	Q1
4	Plos One	19	1,376	72.42	2.6	Q2
6	Neurobiology of Aging	13	837	64.38	3.5	Q2
7	Cells	11	393	35.73	5.2	Q2
7	Frontiers in Pharmacology	11	322	29.27	4.8	Q1
7	Scientific Reports	11	274	24.91	3.9	Q1
10	Current Alzheimer Research	10	493	49.30	1.9	Q3
10	Biochimica Et Biophysica Acta-Molecular and Cell Biology of Lipids	10	551	55.10	3.3	Q2

### 3.5 Authors

**FIGURE 6:**
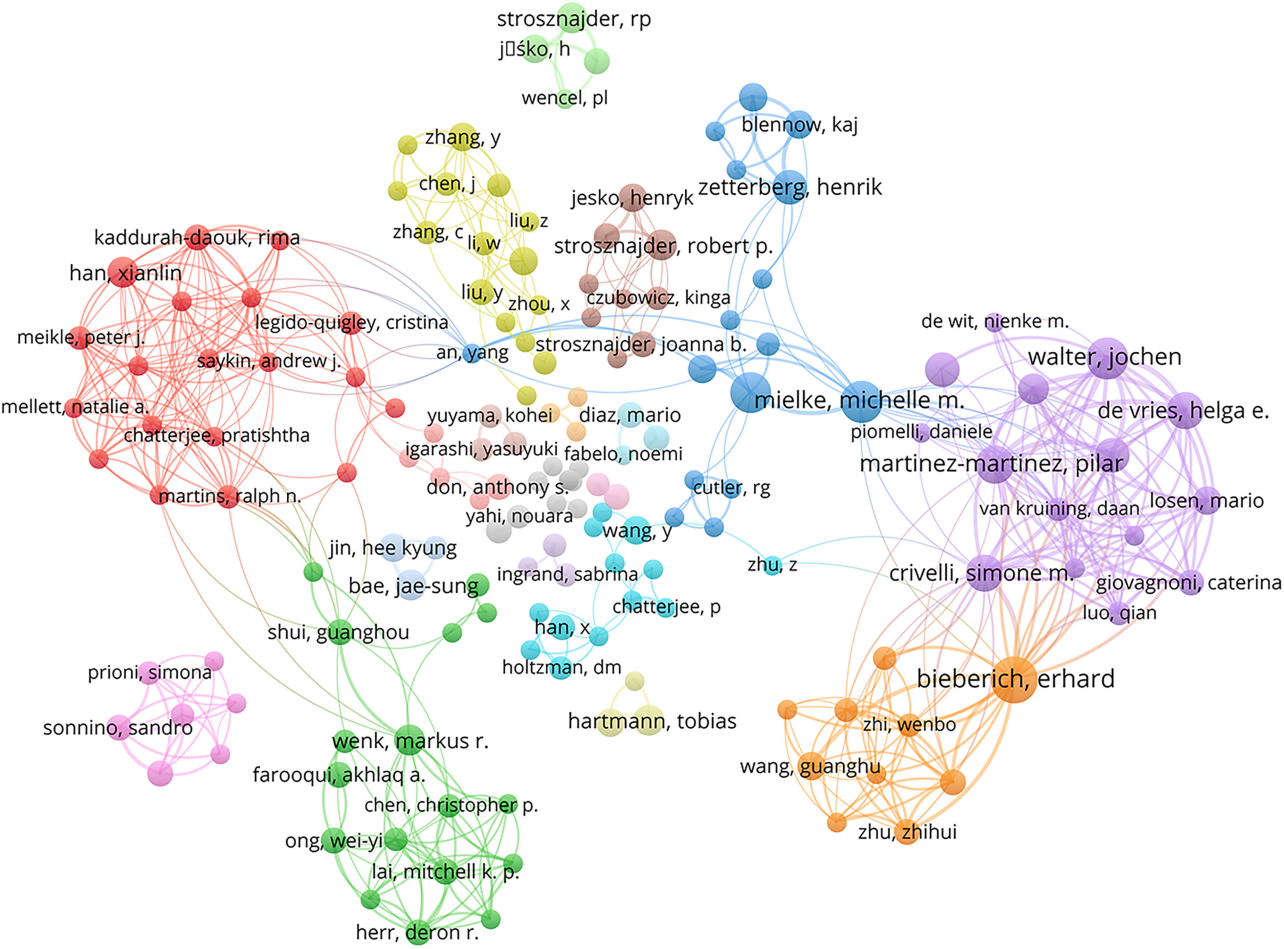
Author collaboration network.

Dr. Erhard Bieberich (16 publications), Dr. Michelle M. Mielke (13 publications), and Dr. Jochen Walter (13 publications) were the three scholars who have published the most in this field and can be regarded as its leaders (Table 4). Researchers from around the world have engaged in extensive collaboration. A combination of small clusters within institutions and large international networks characterized the collaborative model. For instance, Dr. Erhard Bieberich and Dr. Simone M. Crivelli, both from the University of Kentucky in the United States, were close collaborators. Similarly, Dr. Jochen Walter and Dr. Gerhild van Echten-Deckert, both from the University of Bonn in Germany, formed another core team. Cross-border collaboration is widespread, and these small institutional clusters are not isolated. For example, the Walter team from Germany and the de Vries team from the Netherlands, among others, were connected with authors from other countries within the broader collaboration network, reflecting vibrant academic exchange (Figure 6).

**TABLE 4:** The top 10 authors.

Rank	Author	Documents	Country	Institution
1	Dr. Erhard Bieberich	16	USA	University of Kentucky
2	Dr. Michelle M. Mielke	13	USA	Mayo Clinic
2	Dr. Jochen Walter	13	Germany	University of Bonn
4	Dr. Norman James Haughey	12	USA	Johns Hopkins University
5	Dr. Pilar, Martinez-Martinez	11	Netherlands	Maastricht University
6	Dr. Simone M. Crivelli	10	USA	University of Kentucky
6	Dr. Helga E. de Vries	10	Netherlands	Amsterdam UMC
8	Dr. Monique T. Mulder	9	Netherlands	Utrecht University
8	Dr. Gerhild van Echten-Deckert	9	Germany	University of Bonn
8	Dr. Henrik Zetterberg	9	Sweden	Skane University Hospital

### 3.6 Keywords

**FIGURE 7:**
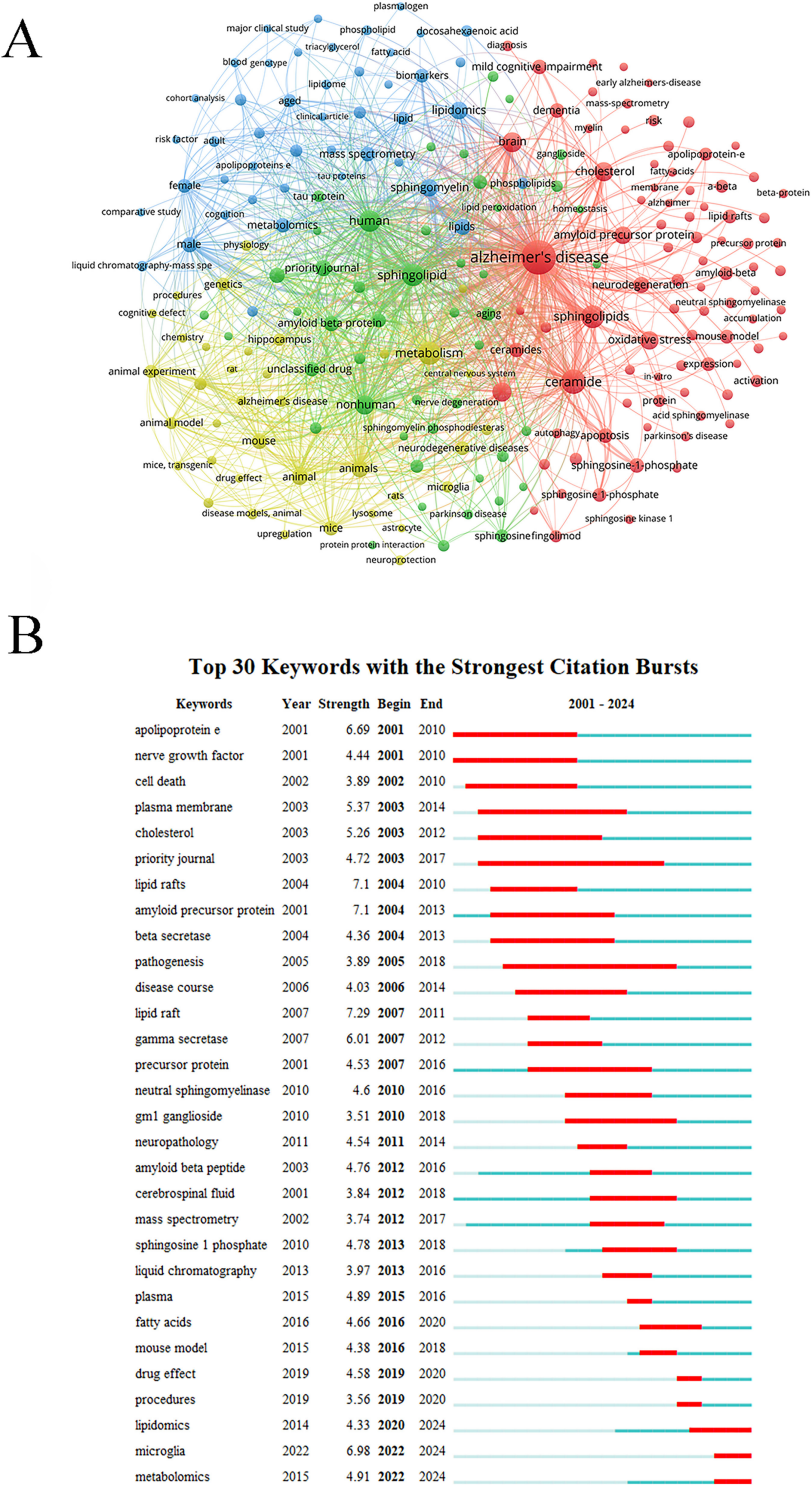
Keywords analysis. (A) Keywords network. (B) Keywords with the strongest citation bursts.

High-frequency keywords effectively illustrate the research focus and hotspots in a given field (Figure 7A). The core research topic was clearly defined: “AD” (frequency: 451; total link strength: 6433) served as the central node of the entire research network. Keywords such as “sphingolipid” (frequency: 141; total link strength: 3,479), “sphingolipids” (frequency: 159; total link strength: 2,300), and “sphingolipid metabolism” (frequency: 98; total link strength: 1,751) indicated that sphingolipid metabolism represented a major research direction in the field of AD. A strong association between sphingolipid metabolism and AD was evident: “ceramide” (frequency: 183; total link strength: 2,805), a key molecule in sphingolipid metabolism, was frequently associated with apoptosis, inflammation, and oxidative stress. “Sphingomyelin” (frequency: 80; total link strength: 1,838), an essential component of cell membranes, may contribute to membrane dysfunction in AD when its metabolism is disrupted. Crossroads of pathological mechanisms were highlighted by terms such as “cholesterol” (frequency: 100; total link strength: 1,478), reflecting broad research interest in lipid metabolism—including both cholesterol and sphingolipids in AD. “Oxidative stress” (frequency: 84; total link strength: 1,021) represented a key pathological process linking AD and sphingolipid metabolism. Meanwhile, “APP” (frequency: 78; total link strength: 1,070) suggested a close connection between Aβ metabolism and sphingolipid metabolism.

From a temporal perspective, burst keywords clearly delineated the evolving research trends and hotspots related to sphingolipids in AD (Figure 7B). The first stage (2001–2010): Keywords such as Apolipoprotein E (APOE), lipid rafts, APP, beta secretase, and cholesterol established the central role of lipid rafts in AD pathology. Researchers have discovered that the gene *APOE*, which causes AD, is related to cholesterol metabolism. Therefore, they focused their research on the lipid microstructure regions rich in cholesterol and sphingolipids—lipid rafts. The second stage (2011–2015): Research shifted from the static structure of lipid rafts to dynamic sphingolipid metabolic enzymes and products. Representative burst terms included neutral sphingomyelinase, sphingosine 1 phosphate, mass spectrometry, and liquid chromatography. The third stage (2015–2020): The research scope was further expanded to cover a wider range of lipid categories. The emergence of the mouse model and drug effect indicated a transition from basic research to preclinical therapeutic interventions. The appearance of plasma reflected efforts to identify blood-based lipid biomarkers for early AD diagnosis. The keyword fatty acids suggested that investigations were extending beyond sphingolipids and cholesterol to encompass a wider lipid metabolic network. The fourth stage (2021–2024): Research hotspots were oriented toward systems biology and neuroimmunology. Lipidomics and metabolomics showed the strongest burst intensity, marking the advent of systematic omics approaches. Microglia emerged as one of the most prominent recent burst keywords, linking neuroinflammation with sphingolipid metabolism, as microglial function is strongly influenced by the lipid microenvironment.

## 4 Discussion

### 4.1 General information

Research on sphingolipids in AD exhibits an overall fluctuating upward trend. The United States and China led in research output in this field. Johns Hopkins University has produced the largest number of publications, while the *Journal of Alzheimer’s Disease* was the most frequently published journal. Dr. Erhard Bieberich was the most prolific author. The collaboration among various global institutions and authors has greatly facilitated the advancement of research in this field.

### 4.2 Hotspots and frontiers

**FIGURE 8:**
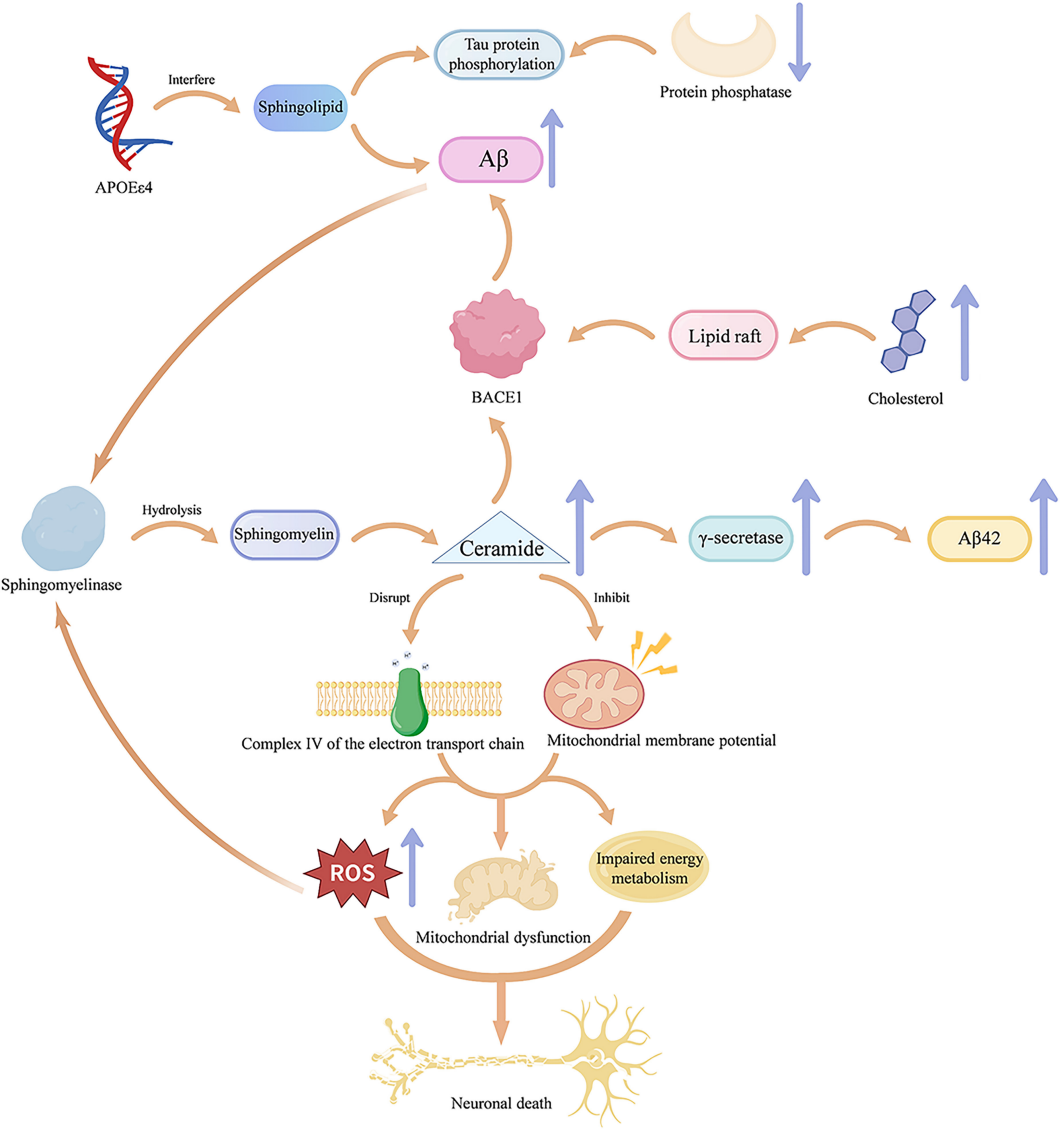
Mechanistic diagram of sphingolipids in AD.

In the context of this research topic, AD represents the core of the entire research network, while sphingolipid metabolism has emerged as a hotspot in AD investigations (Figure 8). Sphingolipid metabolic disturbances are closely associated with the core pathological features of AD, such as Aβ deposition and Tau protein phosphorylation. The strong genetic risk factor *APOE*ε4 exacerbates AD pathology by interfering with sphingolipid metabolism (Fernández-Calle et al., 2022). *APOE*ε4 promotes increased breakdown of sphingomyelin in cerebral blood vessels and enhances ceramide production (Inoue et al., 2025). Ceramides are cytotoxic and can activate related enzymes, further promoting Aβ generation, thereby forming a positive feedback loop that drives disease progression. Studies have shown that ceramide levels are significantly elevated in the brains of AD patients (Filippov et al., 2012). As a pro-apoptotic lipid second messenger, ceramide directly enhances γ-secretase activity, thereby increasing the production of Aβ42, a more toxic form of Aβ. Through mechanisms such as mitochondrial dysfunction and caspase activation, ceramides induce neuronal apoptosis; additionally, by inhibiting protein phosphatase activity, they indirectly lead to hyperphosphorylation of the Tau protein (Jembrek et al., 2015). Sphingomyelin occupies a critical hub position in both sphingolipid metabolism and AD research. It is not only a key component of cell membranes, but its metabolites are also directly involved in the core pathological processes of AD. Sphingomyelin is hydrolyzed by sphingomyelinase, generating ceramide and phosphocholine. In AD, increased activity of sphingomyelinase has been observed, leading to overactivation of the “sphingomyelin-ceramide” pathway, which disrupts homeostatic balance and shifts toward neurotoxicity and inflammation (He et al., 2010). Ceramide produced from sphingomyelin hydrolysis has been shown to promote the activity and stability of BACE1, thereby increasing Aβ production. Aβ oligomers themselves can activate neutral sphingomyelinase (nSMase), resulting in further sphingomyelin hydrolysis and more ceramide generation (Jana and Pahan, 2004). This self-amplifying cycle markedly exacerbates Aβ deposition and neurotoxicity. Oxidative stress, APP, and cholesterol represent major intersecting pathological mechanisms in sphingolipid and AD research. Reactive oxygen species (ROS) and lipid peroxides are potent activators of nSMase. Upon activation, nSMase generates ceramide, representing the most direct link between oxidative stress and sphingolipid metabolism (Jana and Pahan, 2004). Ceramides can form channels in the mitochondrial membrane, disrupt mitochondrial membrane potential, and specifically inhibit complex IV of the electron transport chain. This leads to severe mitochondrial dysfunction, impaired energy metabolism, and explosive ROS production (Gudz et al., 1997). Both high ceramide levels and ROS serve as powerful pro-apoptotic signals, and their combined action irreversibly promotes neuronal death (Cutler et al., 2004). APP cleavage enzymes, particularly BACE1, are predominantly located in lipid rafts. Intracellular cholesterol levels directly determine the quantity, size, and stability of lipid rafts. A high-cholesterol environment promotes lipid raft formation and stability, thereby facilitating the colocalization of APP and BACE1 and significantly enhancing Aβ generation (Simons et al., 1998). Cholesterol and sphingolipid metabolic pathways share organelles (such as endosomes and lysosomes) and interact in both biosynthesis and transport (Nixon, 2005). In early AD, significant abnormalities in the endosome–lysosome system can lead to impaired cholesterol transport and sphingolipid accumulation (Maxfield and Tabas, 2005).

**FIGURE 9:**
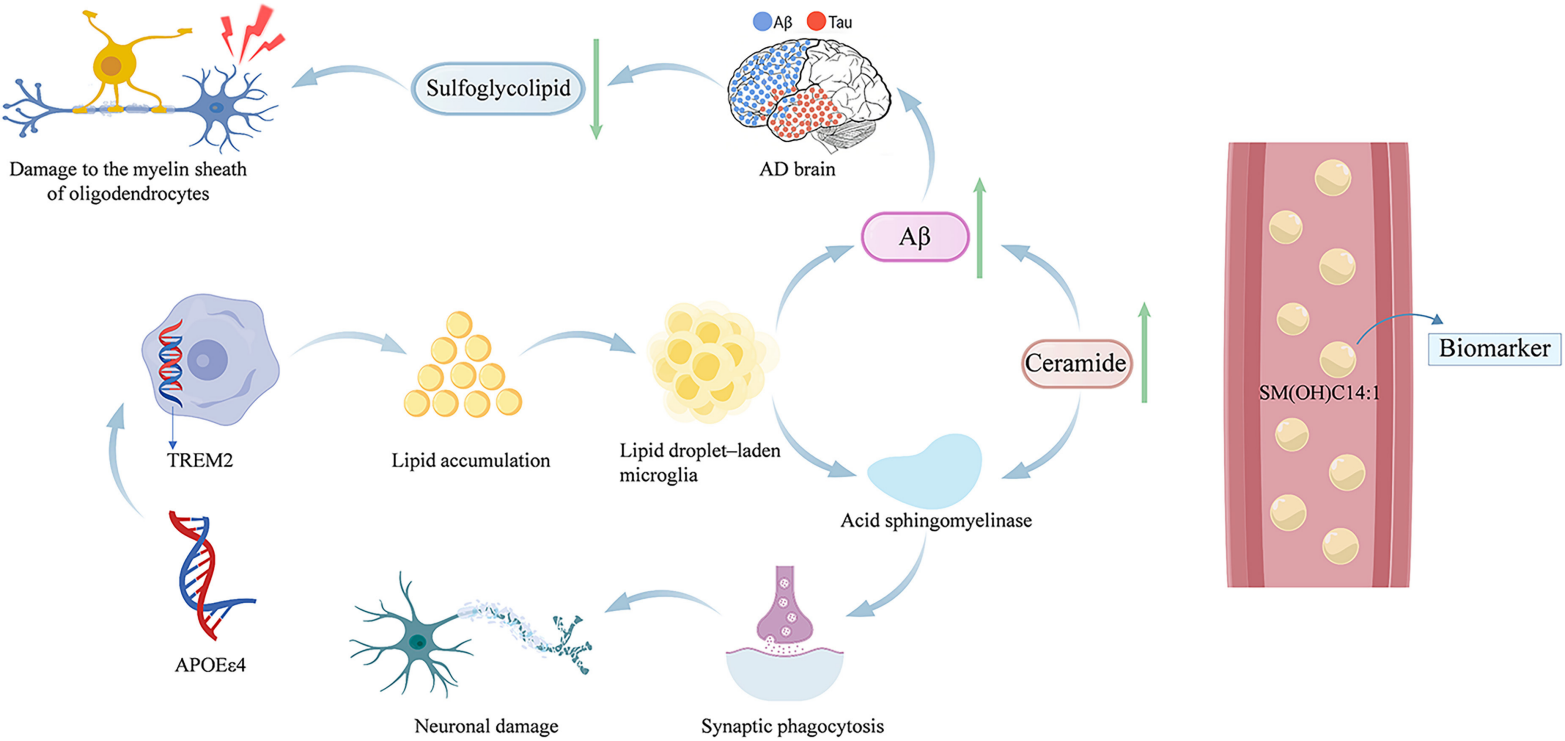
Ceramides, metabolomics, and lipidomics in relation to sphingolipids.

Lipidomics, metabolomics, and microglia are keywords that have exhibited the most pronounced growth in recent years, suggesting they represent future research trends and directions (Figure 9). Lipidomics provides a comprehensive perspective for identifying specific changes in sphingolipid molecules during AD pathogenesis. Lipidomic studies have revealed that sulfoglycolipids, another class of sphingolipids, are significantly and progressively reduced in the brains of AD patients (Palavicini et al., 2022). Sulfoglycolipids are essential components of myelin and cell membranes, and their reduction indicates myelin integrity impairment and oligodendrocyte dysfunction. A lipidomics-based analysis of the cerebral cortex in AD patients demonstrated extensive and progressive lipid metabolic disruptions, with glycerolipid, glycerophospholipid, and sphingolipid pathways being centrally affected (Akyol et al., 2021). A cross-sectional metabolomics study in AD patients revealed that the content of a specific sphingolipid substance, SM(OH)C14:1, was significantly downregulated in the preclinical and early stages of AD (Fote et al., 2021). This suggests that focusing on structurally defined lipid species, rather than broad categories, is more likely to uncover subtle early AD changes. Plasma SM(OH)C14:1 has potential as a predictive biomarker to identify high-risk individuals before clinical symptom onset. In AD, microglial lipids, such as cholesterol and sphingolipids, are central to microglial function. TREM2, a key receptor for microglial sensing and response to Aβ plaques, becomes dysfunctional in AD, disrupting this process and leading to intracellular lipid accumulation. This results in the formation of dysfunctional lipid droplet–laden microglia and exacerbates Aβ pathology (Nugent et al., 2020). The ceramide Cer(d38:1) and phosphatidylserine PS(32:1) are the most significant lipids associated with the risk variations of AD and TREM2 (Proitsi et al., 2026). *APOE*ε4 disrupts the TREM2–APOE signaling axis in microglia and aggravates neuroinflammation in AD by activating the TLR4/NF-κB pathway (Zhang et al., 2025). Activated microglia upregulate acid sphingomyelinase, which further converts sphingomyelin to ceramide, amplifying neurotoxic signaling, promoting synaptic phagocytosis, and causing neuronal damage, thereby impairing learning and memory (Sun et al., 2025). Relevant meta-analysis indicates that some lipid families (fatty acids, glycerides, glycerophospholipids, sphingolipids, lipid peroxidation compounds) show impaired levels in the early stage of AD, with significantly higher levels of ceramides and lower levels of sphingomyelins (Casas-Fernández et al., 2022).

### 4.3 Challenge and opportunity

In early-stage AD patients, levels of specific sphingolipids such as ceramides and sphingomyelin in cerebrospinal fluid and blood undergo marked alterations. These changes offer potential for developing early, non-invasive or minimally invasive blood-based assays, which could help identify at-risk individuals before irreversible brain damage occurs. Despite promising prospects, translating sphingolipid research into clinical practice faces multiple challenges. Sphingolipids constitute not a single molecule, but a large family of hundreds of structurally homologous yet functionally diverse or even antagonistic members. Their distribution, interactions, and dynamic changes within cells are highly complex.

Sphingolipid research offers a broader perspective on AD beyond the conventional focus on Aβ and Tau. It interconnects several core pathological processes, including metabolic dysregulation, neuroinflammation, and neuronal death. Future research may focus on the following directions: utilizing high-throughput omics technologies to establish sphingolipid profiles across different stages of AD; developing highly sensitive sphingolipid blood biomarkers for early screening; designing blood-brain barrier-permeable sphingolipid pathway-targeted drugs that act on specific cell types; and exploring combination therapies targeting sphingolipids along with existing Aβ- or Tau directed drugs.

A cross-sectional study found that plasma ceramides (particularly C18:0 and C24:1) might serve as potential biomarkers for mild cognitive impairment (MCI) in middle-aged men, and there was a significant gender difference (van Kruining et al., 2023). However, due to the cross-sectional design, methodological limitations, and the failure to control key confounding factors (such as APOE), the results need to be further verified in larger-scale, longitudinal studies. Future research should use high-throughput techniques to provide a comprehensive picture of sphingolipid changes over the course of AD. The use of non-targeted lipidomics technology and a systematic analysis of longitudinal cohorts with biomarker confirmation at different stages of AD. Identifying individual sphingolipid subspecies in biofluids that serve as sensitive and specific indicators for early AD screening. Develop drugs that can selectively alter the levels of specific sphingolipid molecules by acting on enzymes with specific acyl chains or head groups. Rational combination of drugs targeting a specific, dysregulated sphingolipid metabolite with Aβ- or Tau-targeting agents, based on converging pathological mechanisms.

This study is based on citation analysis, which has a time lag. The data is up to December 2024, meaning that high-quality and highly influential research results published after this date, due to their citations not having fully accumulated, may not be fully identified or valued in the various indicators of this analysis. This could lead to a certain delay in our capture of the latest research frontiers and may make some earlier published but long-lasting influential works appear more prominent.

## 5 Conclusion

Research on sphingolipids in AD exhibits an overall fluctuating upward trend. Extensive collaboration among researchers from various institutions has facilitated advancements in this field. Sphingolipid metabolism represents a key focus in AD research, with ceramides and sphingomyelins identified as critical molecules. Lipidomics, metabolomics, and microglia are likely to represent future research frontiers.

## Data Availability

The raw data supporting the conclusions of this article will be made available by the authors, without undue reservation.
